# Transcatheter mitral valve replacement to treat rheumatic mitral stenosis: a case series

**DOI:** 10.3389/fcvm.2024.1424105

**Published:** 2024-12-05

**Authors:** Ping Jin, Hong Guo, Yu Mao, Mengen Zhai, Yang Liu, Jian Yang

**Affiliations:** Department of Cardiovascular Surgery, Xijing Hospital, Air Force Medical University, Xi’an, China

**Keywords:** mitral stenosis, rheumatic valvular heart disease, transcatheter mitral valve replacement, prizvalve, mitral valve

## Abstract

**Background:**

Rheumatic mitral stenosis (RMS) is a common valvular heart disease in developing countries. We sought to evaluate the early experience of patients with RMS undergoing transcatheter mitral valve replacement (TMVR).

**Methods:**

In this retrospective study, a total of 5 RMS patients accepted TMVR. All patients underwent computed tomography and echocardiography before having the procedure. After the preprocedural comprehensive evaluations, the surgeons planned to use the Prizvalve (a novel balloon-expandable transcatheter aortic valve system which is now under the clinical registration study) for TMVR. Clinical data were collected at baseline, before discharge, and at the 30-day follow-up.

**Results:**

The median age of the 5 RMS patients was 61 years (range 60–77 years); 60% were male, and the median Society of Thoracic Surgeons score was 13.3% (range 6.2–17.1%). TMVR was successful in all patients. Postoperative transesophageal echocardiography showed that 60.0% (*n* = 3) of the patients had no paravalvular leakage and 40.0% (*n* = 2) had trace paravalvular leakage. The median postoperative peak velocity decreased to 1.4 m/s (range 1.1–1.7 m/s), and the median pressure gradient decreased to 3 mmHg (range 2–3 mmHg). No deaths occurred at the 30-day follow-up, and all patients had an improvement of ≥1 on the New York Heart Association functional rating.

**Conclusions:**

Our early experience with TMVR in RMS patients suggests that it is a safe and feasible procedure. The early results of the procedure are acceptable and provide bright prospects and directions for the precision treatment of RMS.

**Clinical Trial Registration:**

ClinicalTrials.gov, identifier (NCT02917980).

## Introduction

Rheumatic valvular heart disease is one of the major diseases affecting human health around the world. Studies have shown that 33.4 million people worldwide suffer from rheumatic valvular heart disease (1). Rheumatic mitral stenosis (RMS) is particularly common in developing countries (2). At present, the main treatment options for rheumatic mitral valve (MV) disease include MV valvuloplasty and MV replacement (3). Evidence-based studies have shown that, compared with MV replacement, MV valvuloplasty has significant advantages in periprocedural risk and the long-term survival rate (4, 5).

However, for patients with end-stage RMS, MV valvuloplasty is a high-risk procedure with a high likelihood of potential complications. It is worth noting that the development of transcatheter aortic valve replacement in recent years has promoted the exploration of transcatheter mitral valve replacement (TMVR). TMVR largely solves the problem of the ineffectiveness of drug therapy and the high risk of surgical treatment (6, 7). TMVR does not require a thoracotomy and cardiopulmonary bypass and has a better therapeutic effect and prognosis, which offers a new choice for patients with MV disease.

The purpose of this study was to evaluate our early experience using Prizvalve (Newmed Medical Technology Co., LTD., Shanghai, China) for transfemoral TMVR in RMS patients (8). To our knowledge, this is the largest single-center cohort of RMS patients treated with TMVR thus far.

## Methods

### Study population

From September 2022 to June 2023, 5 RMS patients were treated with TMVR at Xijing Hospital. Inclusion criteria included transthoracic echocardiography (TTE), and computed tomography angiography (CTA) confirmed the diagnosis of RMS. In this study, the selection criteria for RMS patients accepting TMVR were as follows: (1) Severe mitral stenosis with orifice area <1.0 cm^2^; (2) Mitral annular calcification; (3) Preoperative LVOT area ≥150 mm^2^; (4) The annulus was observed by CT to estimate the anchoring position and effect. Exclusion criteria included other causes of secondary mitral stenosis, including infective endocarditis, congenital heart disease, and previous left cardiac system surgery. This study was carried out in accordance with the principles of the Declaration of Helsinki and met the relevant ethical requirements. All patients provided written informed consent for TMVR and subsequent data collection.

### Device

**Figure 1:**
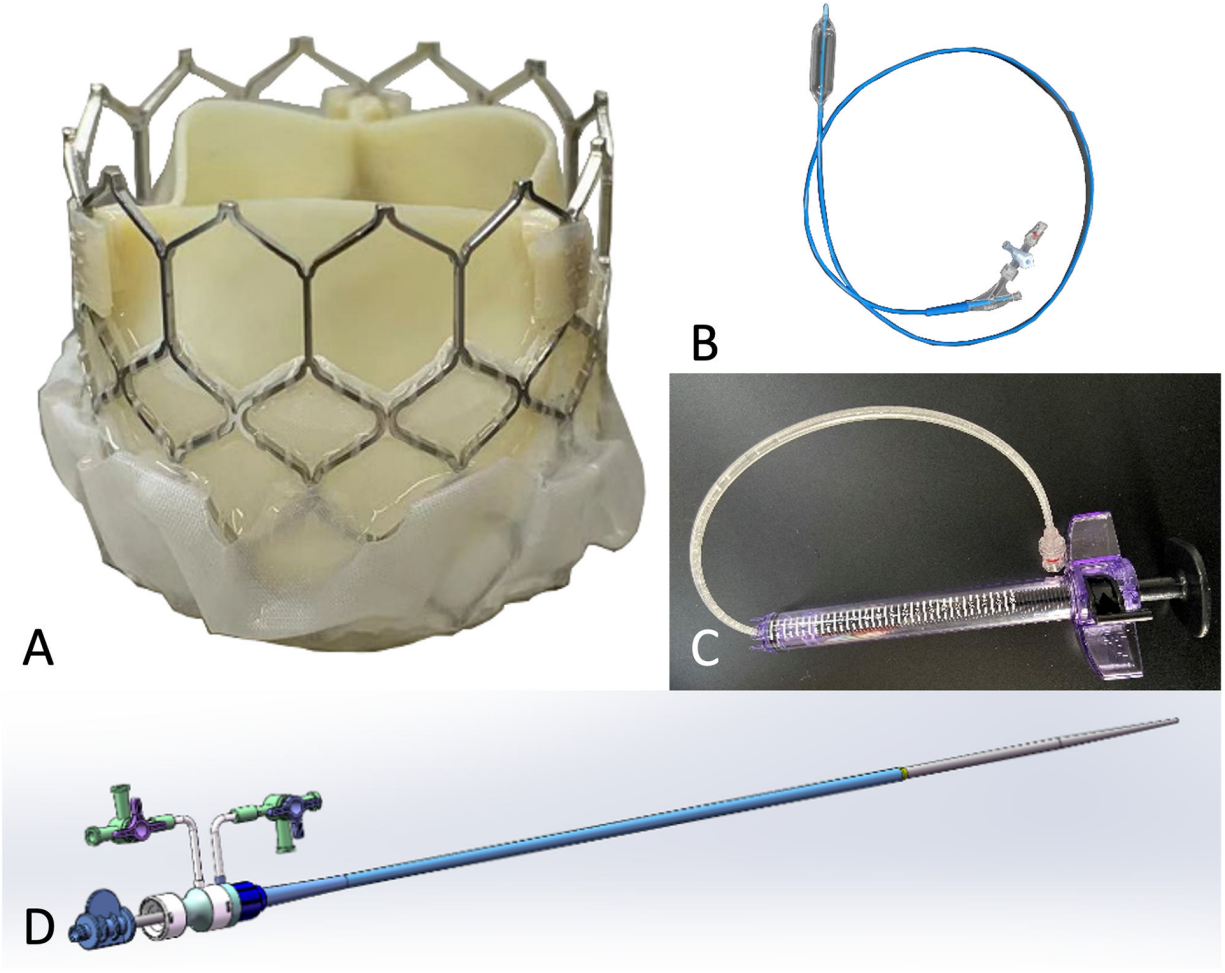
Characteristics of the prizvalve balloon-expandable valve (newmed medical Co., LTD., Shanghai, China). **(A)** Transcatheter heart valve. **(B)** Balloon. **(C)** Balloon pressure pump. **(D)** Expandable arterial sheath.

The Prizvalve balloon-expandable valve (Newmed Medical Co., LTD., Shanghai, China) (Figure 1), made of bovine pericardium, was used in the study. The bottom of the stent valve is covered with a polyester membrane, which can reduce the occurrence of paravalvular leakage. There are three marked points in the middle of the valve that can be clearly seen under fluoroscopy to assist in the accurate positioning of the valve. It is available in 4 sizes: 20 mm, 23 mm, 26 mm, and 29 mm.

### Preprocedural imaging assessment

**Figure 2:**
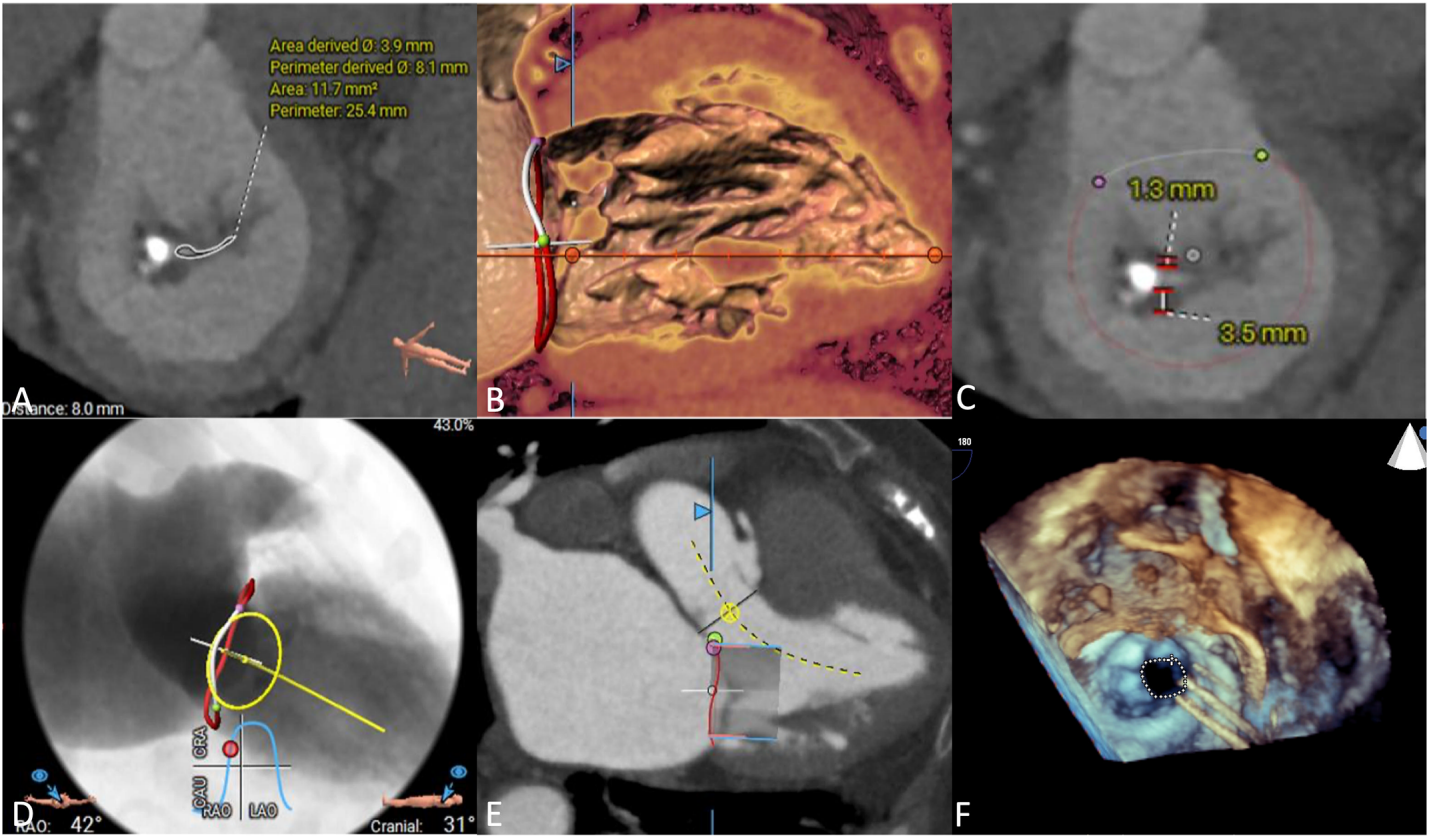
Preprocedural imaging assessments. **(A)** Mitral annulus measurement, the annular area was 11.7 mm^2^. **(B)** Subvalvular apparatus evaluation, chordae tendinae was shown thickened. **(C)** Leaflets of mitral valve evaluation. **(D)** Implanted projection angle determination. **(E)** Neo-left ventricular outflow tract prediction. **(F)** Rheumatic mitral stenosis was displayed in preprocedural echocardiography.

We used CTA to evaluate MV anatomy before the procedures, including evaluation of the mitral annulus, leaflets, left ventricular outflow tract and subvalvular apparatus (Figures 2A–E). Coronary angiography was used to rule out severe coronary artery disease. All patients underwent preprocedural TTE to assess MV and right cardiac system functions (Figure 2F). After assessing each patient's age, frailty, comorbidities, and surgical risk, the cardiac team recommended that all patients undergo TMVR.

### Procedures

**Figure 3:**
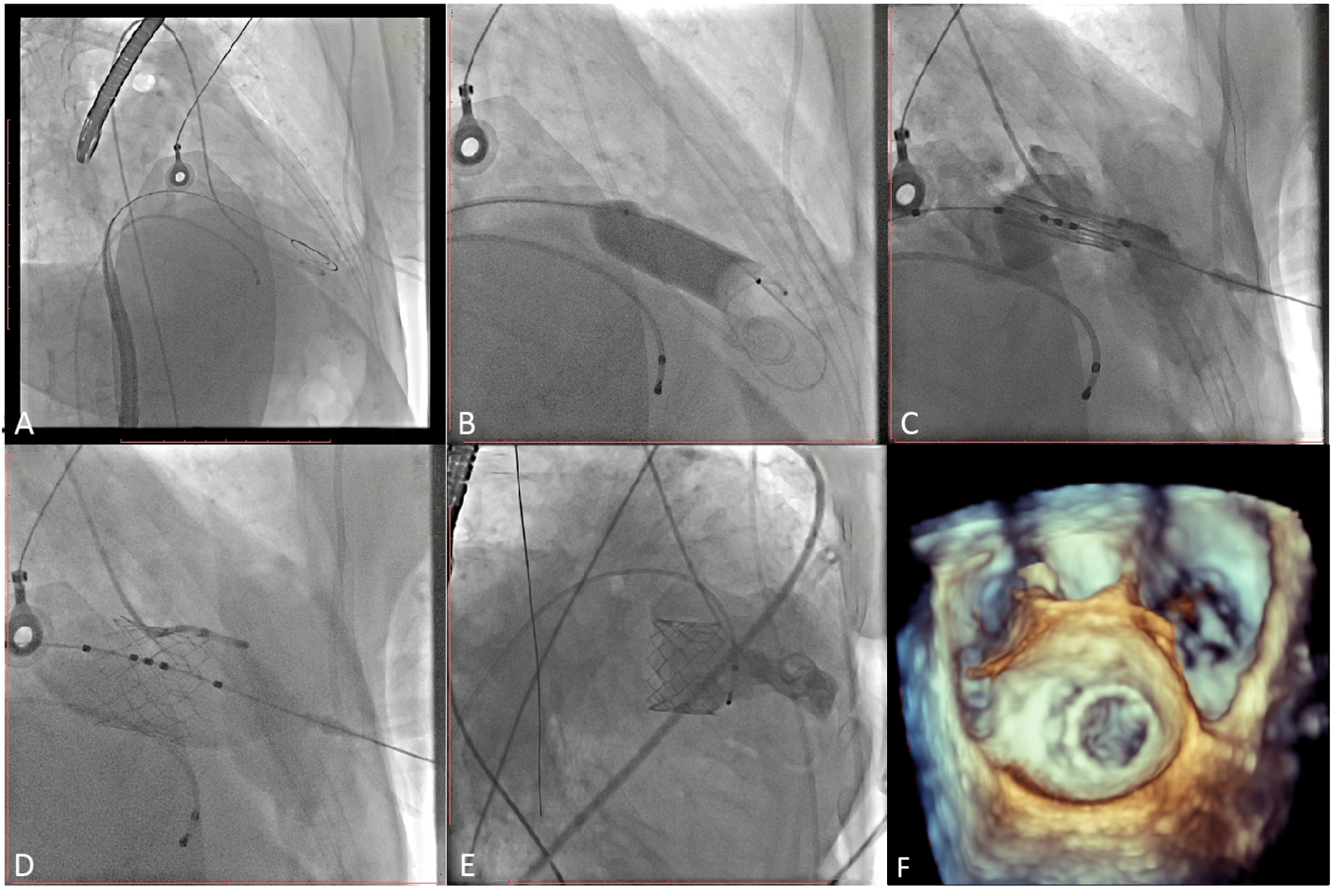
Procedural details. **(A)** The pigtail catheter was inserted into the left ventricle. **(B)** Pre-dilation was used before delivery system insertion. **(C)** The prothesis was positioned at mitral valve. **(D)** The prosthesis was completed release and post-dilation. **(E, F)** Digital subtracted angiography and transesophageal echocardiography displayed the good position and function of the prosthesis.

The patient's bilateral femoral artery region was disinfected; the right femoral vein was selected as the puncture point; and the patient was anesthetized with 2 ml of 2% lidocaine administered via local infiltration. After a successful puncture, a 5 Fr arterial sheath tube was placed, and 3,000 units of heparin was injected intravenously. A 6F sheath tube was inserted through the right common jugular vein and the pacemaker catheter was sent to columnae cordis of right ventricle. Subsequently, a 6 Fr sheath was used to lead the 1.5 m Superstiff guide wire (Boston Science Co. Ltd., Boston, USA) to the left atrium through the atrial septal puncture, and the 6 Fr pigtail catheter was inserted to the left ventricle through the guide wire under digital subtraction angiography (DSA) guidance (Figures 3A,B; Supplementary Videos 1, 2). Then, the mitral annulus was marked, and the Prizvalve delivery system was implanted via the guide wire. Pacing to 180 beats/min, and the Prizvalve prothesis was gradually released (Figure 3C; Supplementary Video 3). After the release was completed (Figure 3D; Supplementary Video 4), DSA (Figure 3E; Supplementary Video 5) and transesophageal echocardiography (TEE) (Figure 3F; Supplementary Video 6) showed that the prosthesis was well fixed without regurgitation or paravalvular leakage (PVL).

### Data collection and statistical analysis

The clinical records of all patients were reviewed. TTE was performed 30 days after TMVR to examine the improvement of cardiac function. In addition, all patients underwent CTA at the 30-day follow-up.

SPSS 26.0 software (IBM Corp, Armonk, NY, USA) was used for the statistical analyses. Continuous variables were reported as the median (25th and 75th percentiles), and categorical variables were expressed as frequency and percentage. A double-tail *P-*value <0.05 was considered statistically significant.

## Results

### Baseline characteristics

The median age of patients with RMS was 61 years (range 60–77 years); 60.0% (*n* = 3) were male; and the median Society of Thoracic Surgeons score was 13.3% (range 6.2–17.1%). Detailed baseline characteristics are shown in Table 1. Patient #2 had a history of stroke, and 2 patients had atrial fibrillation. Importantly, the median Wilkins score was 12 (range 10–12). Before TMVR, all patients were evaluated by an interdisciplinary cardiac team and were considered either unable to undergo MV repair or at higher risk due to comorbidities. The results of preprocedural imaging measurements are listed in Table 2. It is worth noting that the median MV area was 0.6 cm^2^ (range 0.5–0.8 cm^2^), the median peak velocity was 2.7 m/s (range 2.4–3.0 m/s), and the median mean pressure gradient was 9 mmHg (range 7–11 mmHg). All patients had ≥ moderate mitral regurgitation. Importantly, 4 patients (80.0%) had ≥ moderate tricuspid regurgitation and 3 patients (60.0%) had pulmonary hypertension.

**Table 1:** Baseline characteristics.

Characteristics	Patient #1	Patient #2	Patient #3	Patient #4	Patient #5
Age, years	75	61	60	60	77
Sex	Female	Male	Male	Male	Female
Body mass index, kg/m^2^	20.6	24.5	25.2	26.0	21.7
Diabetes mellitus	No	No	No	No	No
Hypertension	Yes	No	No	No	Yes
Dyslipidemia	No	No	No	No	No
Cerebrovascular disease	No	Yes	No	No	No
Peripheral artery disease	No	No	No	No	No
Chronic kidney disease	No	No	No	No	Yes
Coronary artery disease	No	No	No	No	No
Previous PCI	No	No	No	No	No
Previous CABG	No	No	No	No	No
Atrial fibrillation	No	Yes	Yes	No	No
Previously implanted pacemaker	No	No	No	No	No
NYHA functional class	III	III	III	III	IV
STS score,%	14.2	17.1	8.6	6.2	13.3
Wilkins score	12	12	11	10	12

CABG, coronary artery bypass graft; NYHA, New York Heart Association; PCI, percutaneous coronary intervention; STS, Society of Thoracic Surgeons.

**Table 2:** Preprocedural imaging assessment.

Characteristics	Patient #1	Patient #2	Patient #3	Patient #4	Patient #5
Transthoracic echocardiography					
Left ventricular ejection fraction, %	58	52	37	55	53
Mitral annular area, cm^2^	0.7	0.5	0.6	0.6	0.8
Mitral valve peak velocity, m/s	2.4	3.0	2.8	2.7	2.4
Mean mitral valve pressure gradient, mmHg	8	11	10	7	9
Mean left ventricular outflow tract pressure gradient, mmHg	24	29	26	26	25
Combined with ≥ moderate MR	Yes	Yes	Yes	Yes	Yes
Combined with ≥ moderate TR	Yes	Yes	Yes	No	Yes
Combined with pulmonary hypertension	No	Yes	Yes	No	Yes
Computed tomography angiography					
Left atrial left–right diameter, mm	55.27	53.50	57.42	52.68	49.76
Left ventricular diameter, mm					
End-diastolic left ventricle diameter (long axis)	66.14	79.62	74.73	82.27	69.14
End-diastolic left ventricle diameter (short axis)	45.31	46.83	46.58	56.19	50.83
Left-right diameter	46.62	44.57	47.03	57.17	50.50

MR, mitral regurgitation; TR, tricuspid regurgitation.

### Procedural details

**Figure 4:**
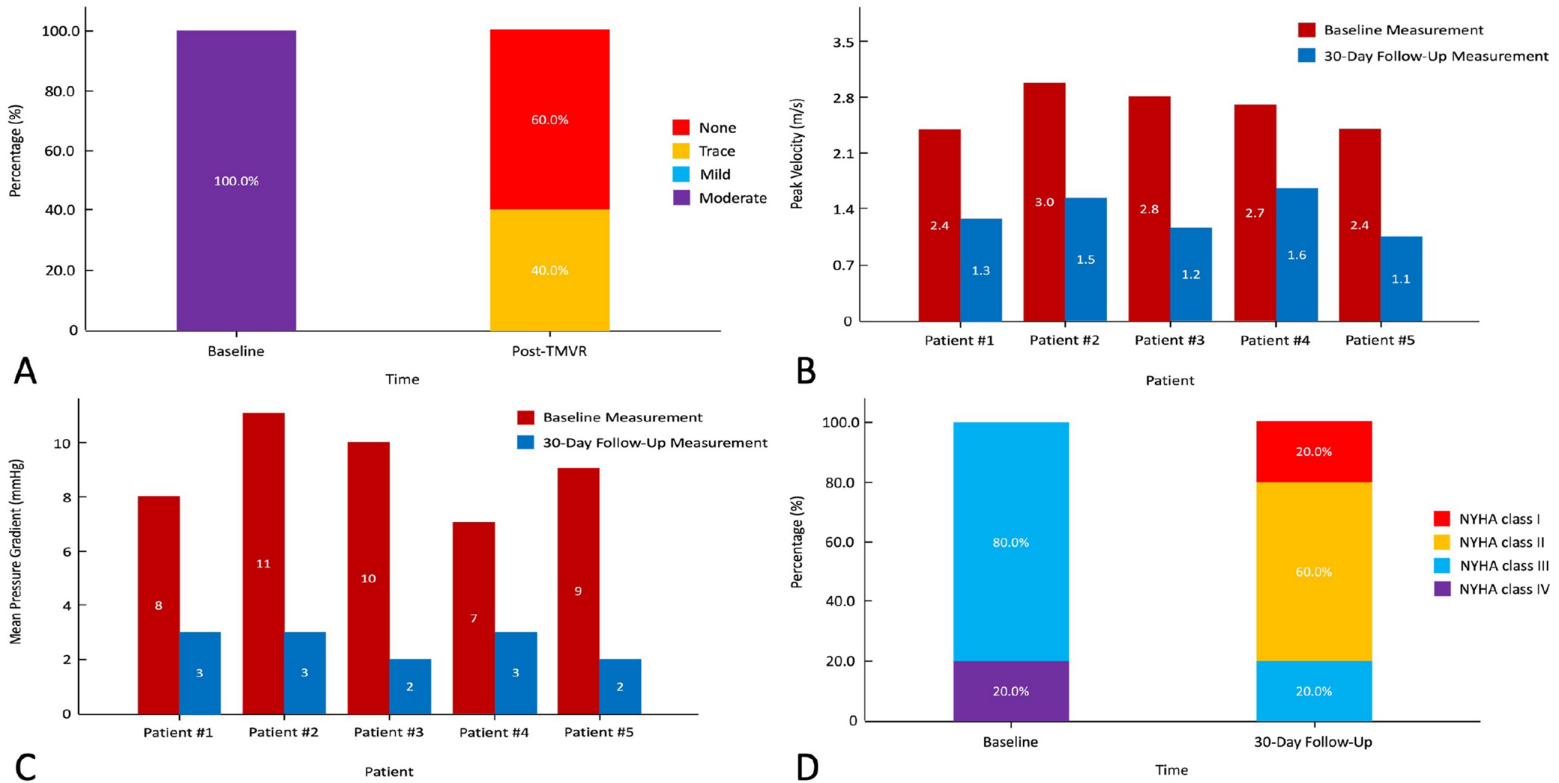
Procedural and follow-up outcomes of patients with rheumatic mitral stenosis. **(A)** Severity of paravalvular leakage. **(B)** Peak velocity of the mitral valve. **(C)** Mean pressure gradient. **(D)** New York Heart Association functional class. NYHA, New York Heart Association.

Detailed procedural outcomes are shown in Table 3. All (100%) patients were successfully implanted with the Prizvalve prosthesis. Two 26-mm and three 29-mm prostheses were implanted according to the mitral annulus diameter measured by CTA and TEE. No patient converted to open surgery, and no major adverse cerebrovascular events occurred. Furthermore, there was no annulus rupture or device displacement. The median total operating time was 170 min (range 85–215 min), the median DSA time was 22 min (range 13–35 min), and the median contrast volume was 118 ml (range 89–142 ml). Postoperative transesophageal echocardiography showed that 60.0% (*n* = 3) of the patients had no PVL and that 40.0% (*n* = 2) of the patients had trace PVLs (Figure 4A). It is worth noting that patient #3 developed severe hypotension (63/37 mmHg) after the procedures and was given extracorporeal membrane oxygenation. As expected, the median postprocedural peak velocity decreased to 1.4 m/s (range 1.1–1.7 m/s) (Figure 4B), and the median mean pressure gradient decreased to 3 mmHg (range 2–3 mmHg) (Figure 4C).

**Table 3:** Procedural details.

Characteristics	Patient #1	Patient #2	Patient #3	Patient #4	Patient #5
Intraprocedural outcomes					
Prosthetic size, mm	29	26	26	29	29
Procedure duration, min	215	205	160	85	170
Fluoroscopy time, min	35	26	19	13	22
Contrast volume, min	142	133	114	89	118
Complications					
Conversion to surgery	No	No	No	No	No
Prosthesis displacement	No	No	No	No	No
Annular rupture	No	No	No	No	No
Paravalvular leakage	Trace	None	Trace	None	None
Third-degree atrioventricular block	No	No	No	No	No
Postprocedural transesophageal echocardiography parameters					
Extracorporeal membrane oxygenation	No	No	Yes	No	No
Left ventricular ejection fraction,%	54	50	35	53	55
Mitral valve peak velocity, m/s	1.4	1.6	1.2	1.7	1.1
Mean mitral valve pressure gradient, mmHg	3	3	2	3	2
Mean left ventricular outflow tract pressure gradient, mmHg	1	4	1	2	2

### Clinical outcomes

The major clinical outcomes and follow-up data are shown in Table 4. Three patients (60.0%) were extubated in the intensive care unit on the first day after the procedure. According to the Acute Kidney Injury Network criteria (9), patient #3 developed stage 3 acute kidney injury. After medication, the patient's symptoms improved significantly, and he recovered before discharge without dialysis. The median length of stay in the intensive care unit and in the hospital was 2 days (range 1–6 days) and 13 days (range 6–22 days), respectively. There were no deaths, no neurological complications (including stroke and transient ischemic attacks), or myocardial infarction/vascular complications during the hospitalization. No patients required permanent pacemaker implants during the periprocedural period. At the 30-day follow-up, one patient (20.0%) was in NYHA Class I and the other 4 patients (80.0%) were in NYHA Class II (Figure 4D). No major adverse events occurred, and 3 patients (60.0%) experienced reverse remodeling of the left cardiac system.

**Table 4:** Hospitalization and follow-up outcomes.

Characteristics	Patient #1	Patient #2	Patient #3	Patient #4	Patient #5
Hospitalization outcomes					
Death	No	No	No	No	No
Major adverse cardiovascular events	No	No	No	No	No
Stroke	No	No	No	No	No
Life-threatening/major bleeding	No	No	No	No	No
Major vascular complication	No	No	No	No	No
Acute kidney injury stage 2 or 3	Yes	No	Yes	No	No
Permanent pacemaker implant	No	No	No	No	No
Extubating time, days	3	1	3	1	1
ICU stay, days	4	1	6	1	2
Hospitalization stay, days	6	7	22	14	13
Follow-up outcomes					
Death	No	No	No	No	No
Major adverse cardiovascular events	No	No	No	No	No
NYHA functional class	II	I	II	II	III
Left ventricular ejection fraction,%	56	52	42	55	56
Mitral valve peak velocity, m/s	1.3	1.5	1.2	1.6	1.1
Mean mitral valve pressure gradient, mmHg	3	3	2	3	2
Mean left ventricular outflow tract pressure gradient, mmHg	2	3	1	2	1
Left atrial left–right diameter, mm	53.43	51.18	57.20	51.65	50.71
Left ventricular diameter, mm					
End-diastolic left ventricle diameter (long axis)	63.89	81.17	71.23	79.88	70.33
End-diastolic left ventricle diameter (short axis)	43.65	48.37	44.79	54.50	51.62
Left–right diameter	43.70	49.63	45.41	53.92	52.08

ICU, intensive care unit; NYHA, New York Heart Association.

## Discussion

The early clinical outcomes of this study found that TMVR with Prizvalve was safe and feasible in patients with RMS. China is ranked second in the world for the incidence of rheumatic valvular disease (1, 10). RMS is characterized by comprehensive pathological changes in the mitral annulus, chordae tendineae, and the papillary muscle at the same time. The pathological changes are relatively complex, resulting in various manifestations of MV dysfunction. Importantly, the lack of calcification of the mitral annulus in patients with RMS may lead to increased difficulty in positioning the prosthesis. Therefore, for the treatment of such patients, ensuring the integrity of the prosthesis morphology plays an important role in maintaining left ventricular function (11).

Unlike transcatheter aortic valve replacement, TMVR devices are more difficult to develop. Due to the asymmetric saddle-shaped MV anatomy, the MV is greatly deformed when the heart is beating, and the pressure gradient is relatively high, so the prosthesis is easy to shift and has the risk of PVL. Furthermore, the MV is adjacent to the left ventricular outflow tract, and the prosthesis may cause left ventricular outflow tract obstruction (12). However, TMVR has several distinct advantages over transcatheter MV repair. First, TMVR is suitable for different types of MV disease and different pathological changes. Second, TMVR has better operability and a higher success rate than transcatheter MV repair. Third, the hemodynamic improvement immediately after the procedure is better and the effect is more stable (13). This study shows that although the long-term follow-up is still in progress, TMVR has demonstrated certain technical advantages in the treatment of elderly patients with RMS who are at high risk and cannot tolerate a thoracotomy.

In our experience, it is crucial to accurately determine the annulus diameter before TMVR. If the size of the prosthesis is not large enough, the anatomical and pathophysiological uniqueness of RMS may lead to an increased incidence of PVL and device displacement. It is worth noting that careful attention should be paid to positioning when the device is implanted. If the device is positioned inaccurately, PVL (14) and/or left ventricular outflow tract obstruction (15) may develop.

This study has some limitations. First, it a single-center study with a small sample size, so more samples need to be accumulated in the future to confirm the safety and efficacy of TMVR. Second, procedures were completed by interventionists. There is a lack of standardized procedures for the selection of device type, prosthetic size, procedural approach, and postprocessing methods related to TMVR, as well as the process of device deployment. The heterogeneity may affect the reliability of the conclusions obtained. In addition, the follow-up period is relatively short; there is a need to explore more long-term follow-up observations. Finally, we did not use the dedicated devices (such as Highlife, Tendyne, and Intrepid, etc.) to treat such patients. The reasons are as follows: First of all, unfortunately, the dedicated devices are not currently being used in China. In this study, we used the PrizValve balloon-expandable valve, and the clinical outcomes with small sample size indicated that the bioprosthesis may allow such patients to obtain a larger effective orifice area after procedures. In addition, based on the clinical outcomes obtained in this study, the clinical development of TMVR using the PrizValve balloon-expandable valve in MS patients may carry out in the next step to further evaluate the clinical safety and efficacy of the device in patients with MS.

## Conclusions

Overall, TMVR may be an alternative treatment for RMS patients with high surgical risk. Our early experience with TMVR in patients with RMS shows that these procedures are feasible and that early clinical outcomes are reliable. The specific anatomical challenges of RMS may require more in-depth analysis to ensure better results. In addition, further sample collection and follow-up are needed to determine the safety and efficacy of this approach.

## Data Availability

The datasets presented in this study can be found in online repositories. The names of the repository/repositories and accession number(s) can be found in the article/Supplementary Material.

## Supplementary material

Supplementary Video 1 The pigtail catheter was inserted into the left ventricle.

Supplementary Video 2 Pre-dilation.

Supplementary Video 3 Position.

Supplementary Video 4 Release.

Supplementary Video 5 Postprocedural digital subtracted angiography.

Supplementary Video 6 Postprocedural transesophageal echocardiography.
